# A Label-Free and Sensitive Fluorescent Qualitative Assay for Bisphenol A Based on Rolling Circle Amplification/Exonuclease III-Combined Cascade Amplification

**DOI:** 10.3390/nano6100190

**Published:** 2016-10-21

**Authors:** Xia Li, Juan Song, Qing-Wang Xue, Fu-Heng You, Xia Lu, Yan-Cong Kong, Shu-Yi Ma, Wei Jiang, Chen-Zhong Li

**Affiliations:** 1 Department of Chemistry, Liao Cheng University, Liaocheng 252059, China; lixia@lcu.edu.cn (X.L.); songjuan19910321@163.com (J.S.); xueqingwang1983@163.com (Q.-W.X.); youfuheng1993@sina.com (F.-H.Y.); luxia2401012932@163.com (X.L.); kongyancong1212@163.com (Y.-C.K.); mashuyi1212@126.com (S.-Y.M.); 2 School of Chemistry and Chemical Engineering, Shandong University, Jinan 250100, China; 3 Nanobioengineering/Bioelectronics Laboratory, Department of Biomedical Engineering, Florida International University, Miami, FL 33174, USA

**Keywords:** bisphenol A, cascade amplification, Exo III, label-free, rolling circle amplification

## Abstract

Bisphenol A (BPA) detection in drinking water and food packaging materials has attracted much attention since the discovery that BPA can interfere with normal physiological processes and cause adverse health effects. Here, we constructed a label-free aptamer fluorescent assay for selective and sensitive detection of BPA based on the rolling circle amplification (RCA)/Exonuclease III (Exo III)-combined cascade amplification strategy. First, the duplex DNA probe (RP) with anti-BPA aptamer and trigger sequence was designed for BPA recognition and signal amplification. Next, under the action of BPA, the trigger probe was liberated from RP to initiate RCA reaction as primary amplification. Subsequently, the RCA products were used to trigger Exo III assisted secondary amplification with the help of hairpin probes, producing plenty of “G-quadruplex” in lantern-like structures. Finally, the continuously enriched “G-quadruplex lanterns” were lightened by zinc(II)-protoporphyrin IX (ZnPPIX) generating enhanced fluorescence signals. By integrating the primary RCA and secondary Exo III mediated cascade amplification strategy, this method displayed an excellent sensitivity with the detection limits of 5.4 × 10^−17^ M. In addition, the anti-BPA aptamer exhibits high recognition ability with BPA, guaranteeing the specificity of detection. The reporter signal probe (G-quadruplex with ZnPPIX) provides a label-free fluorescence signals readout without complicated labeling procedures, making the method simple in design and cost-effective in operation. Moreover, environmental samples analysis was also performed, suggesting that our strategy was reliable and had a great potential application in environmental monitoring.

## 1. Introduction

Bisphenol A (BPA), a monomer for polycarbonate resin and epoxy resin, has been reported to have an estrogenic activity in human beings [1]. It can bind to the estrogen receptors, up-regulate estrogen receptor-mediated gene expression, and induce various diseases, including breast cancer, prostate cancer, birth defect, and infertility [2,3]. Due to the large number of applications of BPA, the exposure sources to human beings are multiple, primarily present in drinking water [4]. It has been demonstrated that a low intake level for BPA can cause adverse health effects; therefore, the Chinese National Standard (GB 5749-2006) proposed that the BPA content of drinking water should not exceed 10 ng/mL. This fuels the need to develop highly sensitive and selective methods for the quantification of BPA in environmental monitoring, food control, and clinical diagnostics.

The commonly used methods for BPA detection are based on liquid chromatography [5], gas chromatography assay [6], electrochemical assays [7], and colorimetric assays [8]. Aside from these methods, fluorescence-based methods are attractive because of their high sensitivity and easy operation [9]. Many fluorescent BPA detection assays have been designed by employing aptamer as the molecular recognition elements [10,11]. However, in the case of low abundance BPA, the sensitivities of the reported fluorescent assays are not entirely satisfactory, so various signal amplification approaches have been introduced into the fluorescent BPA assays [12,13,14]. Among these signal amplification techniques, DNA amplification attracts much attention because of the high signal amplification efficiency. Polymerase chain reaction (PCR) is the most widely used technique for genetic testing and for the detection of low levels of DNA targets [15]. Despite the attractive sensitivity of PCR, it requires large and expensive thermal cyclers, which largely restrict its wide applications in biological studies [16,17]. In response, isothermal amplification of nucleic acids has been developed as a promising alternative in which rapid and efficient amplification is achieved at a constant temperature without the tedious thermo-cycling [18]. Based on the signal amplifying mechanism, isothermal amplification strategies can be classified into three types: linear amplification, exponential amplification, and cascade amplification [19,20,21,22,23,24]. Compared to linear amplification and exponential amplification, cascade amplification assays can provide a higher amplified signal efficiency and a more elegant sensing strategy by integrating two or more amplification methods. With the capacity to accumulate thousands of ssDNA products, strand displacement amplification (SDA) has been widely applied in different cascade amplification strategies. Recently, Zhou et al. [25] developed a SDA/Exonuclease III (Exo III)-combined cascade amplification platform by employing Exo III as a protecting agent for dsDNA Y junction products and employing SYBR Green I as the fluorescent indicator. Although this method achieved a significantly higher amplified signal by the cascade amplification process, a high background signal from nonspecific stains to dsDNA substrate or hairpin probes resulted in unsatisfactory sensitivity in real water samples. Thus, the design of a low background cascade amplification assay for sensitive and selective detection of trace amounts of BPA is high desirable.

Similar to SDA, Rolling circle amplification (RCA) is an advanced isothermal DNA replication process that has the ability to amplify a primer-template recognition event into repetitive sequence units [26,27]. Therefore, RCA-combined cascade amplification will confer a high sensitivity, which is a powerful signal amplification tool to derive amplified biosensors [28]. The basic design of an RCA-combined cascade sensing DNA assay has been adapted in our previously work based on a combination of magnetic nanoparticles (MNPs) and Exo III-induced cascade two-stage isothermal amplification [29]. However, the functionalization of MNPs brings about complexity and high cost. In addition, the slow DNA hybridization and enzyme kinetics in the surface of MNPs restrict its use in wide applications. Thus, the development of a facile, homogeneous, label-free signal-amplified approach is highly desirable.

Herein, we developed a more facile RCA/Exo III-combined cascade signal amplification strategy for highly sensitive and selective detection of BPA. First, the duplex DNA probe (RP) with anti-BPA aptamer and trigger sequence was designed for BPA recognition and signal amplification. Next, under the action of BPA, the trigger probe was liberated from RP to initiate RCA reaction as primary amplification. Subsequently, the RCA products were used to trigger Exo III assisted secondary amplification with the help of hairpin probes (GHP), producing plenty of “G-quadruplex” in lantern-like structures. Finally, the continuously enriched “G-quadruplex lanterns” were lightened by zinc(II)-protoporphyrin IX (ZnPPIX) generating enhanced fluorescence signals. By integrating the primary RCA and secondary Exo III mediated cascade amplification strategy, this method displayed an excellent sensitivity with the detection limits of 5.4 × 10^−17^ M. In addition, the anti-BPA aptamer exhibits high recognition ability with BPA, guaranteeing the specificity of detection. The reporter signal probe (G-quadruplex with ZnPPIX) provides a label-free fluorescence signals readout without complicated labeling procedures, making the method simple in design and cost-effective in operation. Moreover, environmental samples analysis was also performed, suggesting that our strategy was reliable and had a great potential application in environmental monitoring.

## 2. Results and Discussion

### 2.1. The Principle of the RCA/Exo III-Combined Cascade Signal Amplification Platform for BPA Assay

**Figure 1:**
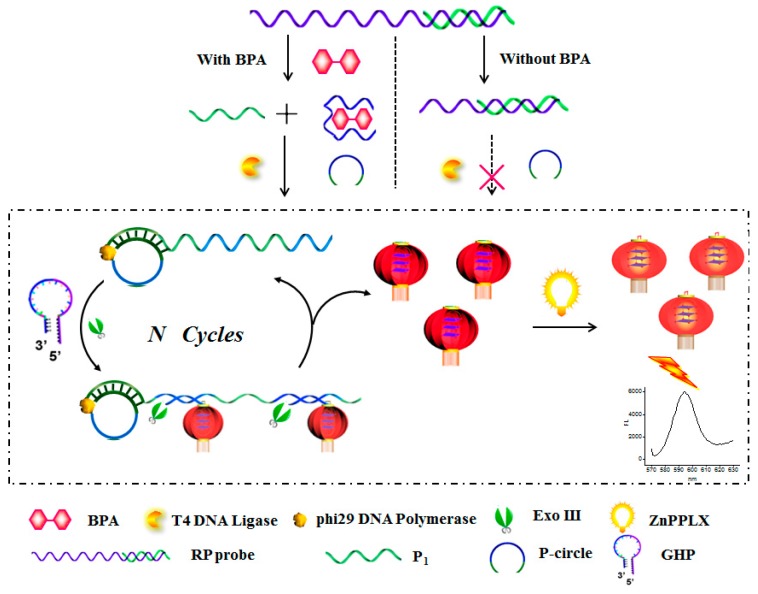
Schematic illustration the principle of the fluorescent assay of Bisphenol A (BPA) based on the rolling circle amplification (RCA)/Exo III-combined cascade signal amplification strategy.

Figure 1 outlines the principal design of the proposed strategy for BPA assay. The DNA duplex strands (recognition probe, denoted as RP) utilized here include the containment of the anti-BPA aptamer (in dark blue) and trigger sequence (denoted as P_1_, in green). In the presence of BPA, the anti-BPA aptamer prefers to switch its configuration to combine with the BPA, releasing trigger strand P_1_, which could anneal to a circular DNA template (P-circle) to form a long DNA tile with multiple recognizing regions for the hairpin probe (in blue). To avoid sophisticated chemical labeling, a G-quadruplex sequence was grafted at the end of GHP (in purple). Upon interaction with the long single DNA bands, multiple GHP were opened simultaneously to form a duplex structure with plenty of “active G-quadruplex” in lantern-like appending on the DNA bands. Subsequently, Exo III is introduced to digest the 3′-end of the resulting duplex structure of the “G-quadruplex lantern”, releasing the DNA ties to trigger the next round of hybridization and cleavage reaction. Finally, ZnPPIX was added in the reaction assay to form G-quadruplex-ZnPPIX complex generating enhanced fluorescence signals. On the contrary, in the absence of BPA, the RP probe is intact, blocking the release of P_1_ and unable to trigger the proposed cascade signal amplification process, thus providing a zero-background signal. On the base of it, the RCA/Exo III-combined cascade signal amplification strategy can be accomplished.

### 2.2. The Verification of the Designed Sensing System

**Figure 2:**
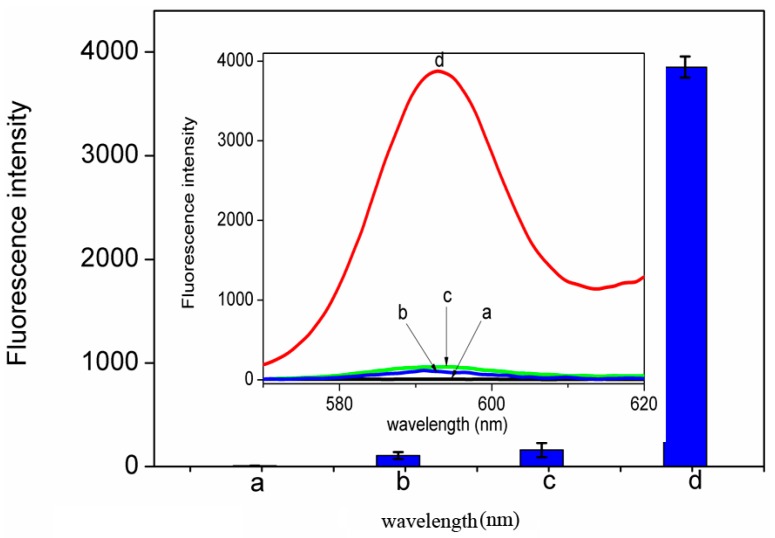
Fluorescence-emission spectra of zinc(II)-protoporphyrin IX (ZnPPIX)/G-quadruplex supramolecular fluorescent labels under different conditions: (**a**) Buffer; (**b**) Buffer + ZnPPIX; (**c**) RP (DNA duplex probe) + Circle DNA + hairpin probes (GHP) + Exonuclease III (Exo III) + ZnPPIX; (**d**) BPA + RP + Circle DNA + GHP + Exo III + ZnPPIX; C_BPA_ = 1.0 μM, C_RP_ = 1.0 μM, C_Circle DNA_ = 100 nM, C_GHP_ = 25 μM, C_Exo III_ = 100 U, C_ZnPPIX_ = 20 μM, RCA reaction time 1.5 h.

As shown in Figure 2, fluorescence measurement was used to investigate the viability of our approach. The system only containing buffer (curve a) or free ZnPPIX (curve b) exhibited a very weak fluorescence intensity. The system containing all assay reagents excluding the target BPA also exhibited slightly enhanced fluorescence (curve c), indicating the RP cannot be opened without target BPA, which impedes the process of the RCA reaction. In this case, the formation of G-quadruplex was difficult when the sequence was partly hidden in the stem of GHP, guaranteeing a zero-background signal. Upon the addition of BPA to the system, further enhanced fluorescence was observed (curve d). It is because the aptamer-BPA binding event leads to the displacement of the primer and initiation of the RCA/Exo III-combined cascade signal amplification event. Thousands of DNA triggers are regenerated, and each of these can open lots of hairpin probes to generate a cascade amplified optical signal. Due to specific recognition between G-quadruplex and ZnPPIX, background reduction and label-free signal amplification were successfully achieved, confirming the feasibility of the proposed strategy. It resulted in a much larger signal to background noise ratio, assuring a higher sensitivity for detection of BPA.

**Figure 3:**
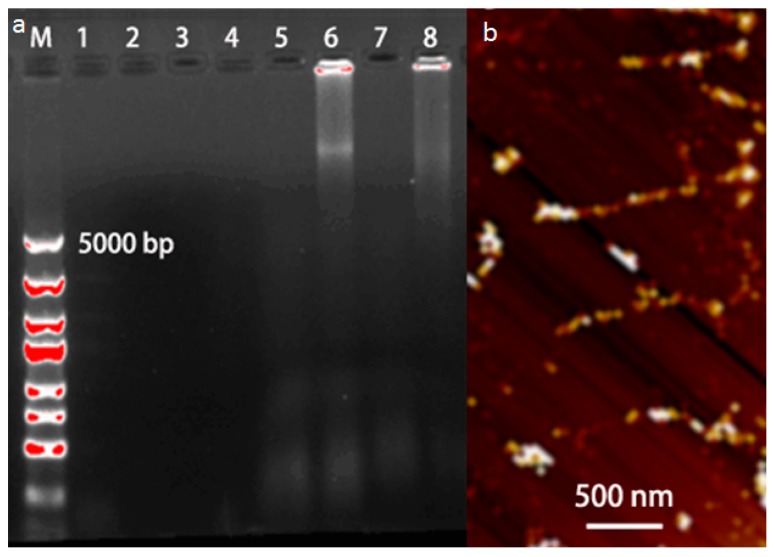
(**a**) Agarose gel (0.7%) electrophoresis: (1) DNA_1_ alone; (2) P_1_ alone; (3) Circle DNA alone; (4) GHP alone; (5) RP + Circle DNA; (6) BPA + RP + Circle DNA; (7) RP + Circle DNA + GHP; (8) BPA + RP + Circle DNA + GHP; (**b**) Atomic force microscope (AFM) images of amplification products of RCA/Exo III-combined cascade signal amplification reaction. C_DNA1_ = 1.0 μM, C_P1_ = 1.0 μM, C_RP_ = 1.0 μM, C_Circle DNA_ = 100 nM, C_GHP_ = 25 μM, C_Exo III_ = 100 U, RCA reaction time 1.5 h.

To further evaluate the amplification products of RCA/Exo III-combined cascade signal amplification reaction, we performed the agarose gel electrophoresis analysis (Figure 3a) and atomic force microscope (AFM) characterization (Figure 3b). As shown in Figure 3a, lanes 1–4 correspond to the anti-BPA aptamer DNA_1_, P_1_, circle DNA, and GHP, respectively, which displayed no bands because of their low molecular weight. Similarly, gel lanes 5 and 7 displayed no bands indicating that the primer P_1_ probe cannot be released in the absence of target BPA, which impeded the process of RCA reaction. Upon the addition of BPA, two bright product bands with high molecular weight could be seen in gel lanes 6 and 8. These results demonstrated that upon recognizing and binding with BPA, the anti-BPA aptamer folds into a closed configuration accompanied by a release of the primary primer. Then, the primary primer strand as amplification units triggered RCA reaction, forming a long DNA band. In addition, the amplification products in our design system were further characterized by AFM. As shown in Figure 3b, multiple DNA bands with lengths of several hundred nanometers up to a few micrometers could be seen, a result which was identical to the fluorescence emission spectra of the system. Therefore, these results demonstrated that the proposed strategy could be adopted for BPA detection.

### 2.3. Optimizing Parameters to Improve the Assay Performance

To achieve the best performance of the proposed assay, we investigated several experimental parameters, such as the probe concentration, Exo III concentration, and the reaction time. Since the aptamer-target binding event is the key to achieve the displacement of the primer and initiation of the RCA process, the effect of the RP probe (aptamer DNA_1_/P_1_) on the response of the biosensor was first evaluated by detecting 100 nM BPA. As shown in Figure S1 (see the Supplementary Materials), the fluorescence signal increases rapidly with the concentration of RP probe from 0.05 µM to 1.4 µM, and reaches a maximum at 1.0 µM, followed by a decrease beyond the concentration of 1.0 µM. Thus, a 1.0 µM recognition probe is used in the subsequent research.

It was widely accepted that the concentration of the circular template and RCA reaction time both had an important effect on the RCA process. As shown in Figure S2a, the fluorescence signal increases rapidly with the concentration of the circular template from 20 to 150 nM and reaches a maximum at 100 nM, thereafter, the fluorescence response exhibited a gradual decrease. This was probably because a large excess of circular templates disturbed their hybridization with the primer in a head-to-tail fashion and due to the subsequent RCA process. As a result, the circular template concentration of 100 nM was selected for further investigation. In addition, the effect of the RCA reaction time on the fluorescence signal was also examined, which is shown in Figure S2b. The fluorescence intensity enhanced quickly with the increase in reaction time, and nearly reached a plateau after 120 min. This might be attributed to the fact that the RCA reaction had reached equilibrium caused by exhaustion of the RCA substrates or inactivation of phi29 DNA polymerase. Therefore, 120 min was chosen as the optimum time for the RCA reaction.

Another factor taken into account for parameter optimization is the GHP probe concentration, since it directly affects the fluorescence readout signal of G-quadruplex. Additionally, the effect of the GHP probe concentration was investigated from 5 μM to 30 μM. As shown in Figure S3, the continuously increasing signal before 20 μM indicates that the designed sensing assay was indeed taking place. The fluorescence response exhibited a gradual decrease with a further increase in the concentrations of the GHP probe, which was probably because a large excess of GHP probes disturbed their hybridization with the RCA products. As a result, the 20 μM GHP probe was selected in the following assay.

We further investigated the influence exerted by the amount of Exo III upon the enrichment efficiency of “G-quadruplex” signal probes. As shown in Figure S4, the fluorescence signal increases rapidly with the exonuclease III concentration from 25 to 200 U and reaches a plateau at 125 U, indicating the highest enrichment efficiency of “G-quadruplex”. As such, the detectable signal mainly derives from the ZnPPIX/G-quadruplex. As such, the concentration of ZnPPIX was also optimized. Experiments indicated that 25 μM of ZnPPIX could provide maximum S/N ratio for the system (see Figure S5, Supplementary Materials).

### 2.4. Detection Performance of the Assay

**Figure 4:**
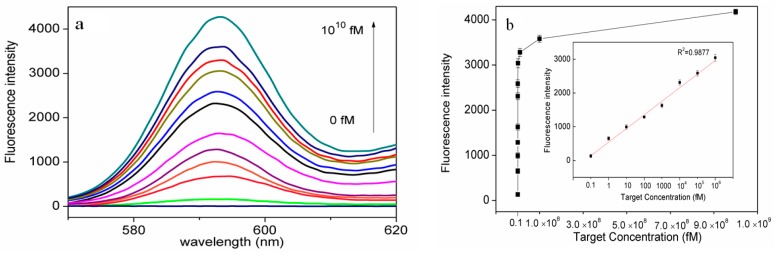
(**a**) Fluorescence emission spectra obtained in the proposed sensing strategy for detection of target BPA with varying concentrations from 0 to 10^1^^0^ fM; (**b**) Calibration curve of fluorescence intensity changes at 595 nm as a function of the concentration of target BPA. Each data point represents an average of three measurements (each error bar indicates the standard deviation).

Under the optimal conditions, the sensitivity and dynamic range of our designed sensing system was investigated using different concentrations of target BPA. As shown in Figure 4a, the fluorescence intensity increased when the concentration of target BPA was raised from 0 to 10^10^ fM, indicating that the liberation of the “caged” inactive G-quadruplex structure in the GHP is highly dependent on the concentration of target DNA. This confirms the working principle that target BPA is hybridized with the recognition probe to displace the primer and initiate the RCA event, further triggering the enrichment of “G-quadruplex” events. Figure 4b showed the fluorescence signal intensity of cascaded ZnPPIX/G-quadruplex complex as a function of the concentration of target BPA. Under the optimal conditions, the fluorescence intensity at the maximum emission wavelength was found to exhibit a linear correlation to the logarithm of target BPA concentrations from 1 nM to 0.1 fM with a linear correlation coefficient of 0.987. According to the three times standard deviation principle, a detection limit of 5.4 × 10^−17^ M was obtained, which was comparable or superior to the most of the previous reported optical amplification methods for BPA detection (Table S1) [19]. The high sensitivity of our approach was probably attributed to the signal amplification of the designed RCA/Exo III-combined cascade signal amplification reaction, as well as the background reduction of the unique dsDNA probe. Hence, the proposed strategy exhibited great promise as an effective tool for qualitative detection of BPA.

The precision and reproducibility of the fabricated fluorescence BPA biosensor were further examined. To evaluate the precision of the proposed strategy, a series of three repetitive experiments of target samples were carried out on the same day. The relative standard deviations (RSD) achieved for the samples containing 0.1 fM and 1 nM of target BPA were 4.2% and 4.7%, respectively. Similarly, a series of three repetitive experiments of target samples were conducted on three different days to demonstrate the repeatability of the strategy. The RSD achieved for samples containing different BPA concentrations (0.1 fM and 1 nM) were 5.6% and 4.9%, respectively. These results indicated that the as-proposed strategy displayed acceptable precision and reproducibility.

### 2.5. Selectivity and Real Sample Analysis

**Figure 5:**
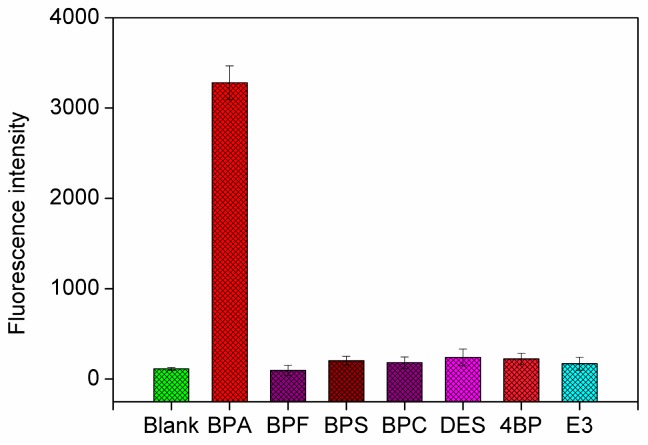
Fluorescence intensity responses at 10 nM of target BPA over other control molecules (200 nM), including BPA, bisphenol F (BPF), bisphenol S (BPS), diethylstilbestrol (DES), 4,4′-biphenol (4-BP), and estriol (E3). Each data point represents an average of three measurements (each error bar indicates the standard deviation).

The capability of this proposed method to perform in real sample analysis conditions was further investigated by testing the fluorescence response of six other control molecules, including bisphenol S (BPS), bisphenol C (BPC), bisphenol F (BPF), 4,4′-biphenol (4-BP), diethylstilbestrol (DES), and estriol (E3), which may coexist in the environment or have similar structures with BPA. As shown in Figure 5, the fluorescence intensity of six control molecules (200 nM) was only about 14% of that for BPA (10 nM). This high specificity could be attributed to the high affinity and specificity of anti-BPA aptamer to BPA, which further ensures a low matrix effect in the practicality of the proposed strategy.

The feasibility of the designed sensing system for practical applications was also investigated by analyzing two environmental water samples (mineral water and tap water) and a general plastic bottle sample. Water samples were spiked with different concentrations of BPA (0, 0.005, 0.01, and 0.015 pM), which were determined with three replicates. The plastic bottle sample was spiked with three different concentrations of BPA (0, 0.036, 0.072, and 0.108 pM), which were determined with three replicates. The recovery results of BPA in the above matrixes are shown in Table S2 (Supplementary Materials). The results in Table S2 show that the sensing platform was affected when target BPA existed in complex matrixes. The results also definitely illuminated the potential application of this sensing system in practical samples. Therefore, the proposed fluorescence biosensor can be successfully applied to BPA detection in real environmental water samples or plastic samples. In addition, the proposed strategy can also be developed as a versatile approach for the sensing of other target molecules only by substituting the target-specific aptamer sequence.

## 3. Materials and Methods

### 3.1. Chemicals

All the HPLC-purified oligonucleotide sequences were synthesized by Shanghai Sangon Biotechnology Co., Ltd. (Shanghai, China) and listed below: the anti-BPA aptamer with the sequence of 5′-CC GGT GGG TGG TCA GGT GGG ATA GCG TTC CGC GTA TGG CCC AGC GCA TCA CGG GTT CGC ACC-3′ (denoted DNA_1_); the 20-base complementary fragment (denoted P_1_), 5′-CCC ACC TGA CCA CCC ACC GG-3′; Circular DNA template (P-circle), 5′-P-GTC AGG T GGG C TTG ATA ACT ACC CGT GAG GAA CCA ATT ATC CCA ACT TAT TCG TGA CTC CAG TGA AGC TAA ATG C CGG TG G GTG-3′; the hairpin DNA probe (GHP), 5′-GGG TTG GGC GGG ATG GGT TAC CTC AGT GCT TAT TCA ACC CTA TTA-3′; Protoporphyrin IX zinc(II) (ZnPPIX) was purchased from Sigma-Aldrich (Shanghai, China) and used without further purification. Phi29 DNA polymerase, T4 RNA Ligase, and dNTP were obtained from Fermentas (Lithuania, Shanghai, China). Exonuclease III (Exo III) was purchased from New England BioLabs (Ipswich, MA, USA). BPA was dissolved with methanol as the stock solution and diluted using the buffer solution for analysis. Other chemicals (analytical grade) were obtained from standard reagent suppliers. Water (18.2 MΩ) was used and sterilized throughout the experiments.

### 3.2. Apparatus

All the fluorescence measurements were performed on a Hitachi F-7000 spectrofluorimeter (Hitachi, Tokyo, Japan). The excitation wavelength was 418 nm, and the spectra were recorded between 570 and 660 nm. The fluorescence emission intensity was measured at 595 nm.

All the AFM measurements were conducted using a Multimode 8 AFM with a Nanoscope V Controller (Bruker Corporation, Beijing, China) in ScanAsyst mode. The measurements were performed in air using a SCANSYST-AIR probe supplied by Bruker Corporation, (Beijing, China).

### 3.3. Procedures for DNA Assay

The detailed procedure for BPA detection was as follows. First, 10 µL of 1 µM DNA_1_ was mixed with 10 µL of 1 µM P_1_ solution in 10 µL of 0.02 M Tris-HCl buffer (MgCl_2_ 20 mM, KCl 40 mM, NaCl 100 mM, pH 8.0) with gentle shaking for 2 min and was incubated for about 30 min at 37 °C. To the above resulting mixture was added 10 μL of BPA standard solution with different concentrations followed by shaking for 2 min and incubating at 37 °C for 30 min. Then 100 nM circular DNA was added, which was heated at 90 °C for 5 min and incubated at 37 °C for 30 min. Then 1 μL of T4 DNA ligase and 1× ligation buffers (400 mM Tris-HCl, pH 7.8, 100 mM MgCl_2_, 100 mM DTT, 5 mM ATP) were added and incubated at 37 °C for 1 h. After ligation, T4 DNA ligase was inactivated by heating the reaction mixture to 65 °C for 10 min. Then, the resulting product was mixed with 1× reaction buffer (330 mM Tris-acetate, 100 mM Mg(Ac)_2_, 660 mM potassium acetate (KAc), 1% Tween-20, and 10 mM DTT (pH 7.8)), 1.0 μL (10 U/μL) Phi29 polymerase and 8 μL 1 μM dNTPs solution. The reaction mixture was incubated at 37 °C for 90 min and terminated by heating 90 °C for 5 min to inactivate the RCA process. After that, the Exo III-assisted signal amplification was performed by incubating the above resulting mixture with 25 μM of GHP and 100 U of Exo III at 37 °C for 90 min. Next, 20 μM ZnPPIX solution was added to the mixture and made the last volume of the solution which was 100 μL. Finally, the mixture solution was incubated at room temperature for 10 min. The fluorescence intensity and the AFM measurement of the mixture solution were measured at room temperature.

### 3.4. Analysis of Actual Water Samples

The reliability of the sensor in practical applications was measured by determining the recovery rate in real water samples and plastic bottles. Tap water samples were obtained from our laboratory. Mineral water and plastic bottles of polyvinyl chloride (PVC) were obtained from a local market. Prior to analysis, the water samples were filtered through a 0.22 µm membrane to remove the insoluble impurities. Then, the water samples were spiked with different concentrations of BPA and diluted five times with the reaction buffer. Three duplicate measurements were performed for all samples. The plastic bottle was sheared into pieces and added into double distilled water and kept for ultra-sonication for 2 h. Then it was kept in a constant temperature water bath for 48 h at 80 °C. Finally, the solution was filtered and kept in a flask. This process was repeated three times and the solution was used as stock solution for further experimentation.

## 4. Conclusions

We have developed a sensitive and selective fluorescence platform for BPA detection using a facile RCA/Exo III-combined cascade signal amplification strategy. This strategy employed a duplex DNA probe to release a trigger strand after being recognized by BPA and the released trigger strand could initiate the proposed cascade signal amplification reaction. By integrating the primary RCA and secondary Exo III mediated cascade amplification strategy, this method displayed an excellent sensitivity with the detection limits of 5.4 × 10^−17^ M. Meanwhile, this approach exhibited exquisite selectivity for BPA by using an anti-BPA aptamer as a sensing element with its high affinity and specificity. In addition, the reporter signal probe (G-quadruplex with ZnPPIX) provided a label-free fluorescence signals readout without complicated labeling procedures, making the method simple in design and cost-effective in operation. Moreover, environmental samples analysis was also performed, suggesting that our strategy was reliable and had a great potential application in environmental monitoring.

## Supplementary Materials

The following are available online at www.mdpi.com/2079-4991/6/10/190/s1.
