# Supplementary material for: A Label-Free and Sensitive Fluorescent Qualitative Assay for Bisphenol A Based on Rolling Circle Amplification/Exonuclease III-Combined Cascade Amplification
Source: Nanomaterials (Basel). 2016 Oct 21;6(10):190. doi: 10.3390/nano6100190 (PMC5245189; doi:10.3390/nano6100190)
Supplement: Supplementary file 1 [file nanomaterials-06-00190-s001.pdf]

Supplementary Materials: A Label-Free and Sensitive Fluorescent Qualitative Assay for Bisphenol A Based on Rolling Circle Amplification/Exonuclease III-Combined Cascade Amplification

Xia Li, Juan Song, Qing-Wang Xue, Fu-Heng You, Xia Lu, Yan-Cong Kong, Shu-Yi Ma, Wei Jiang and Chen-Zhong Li

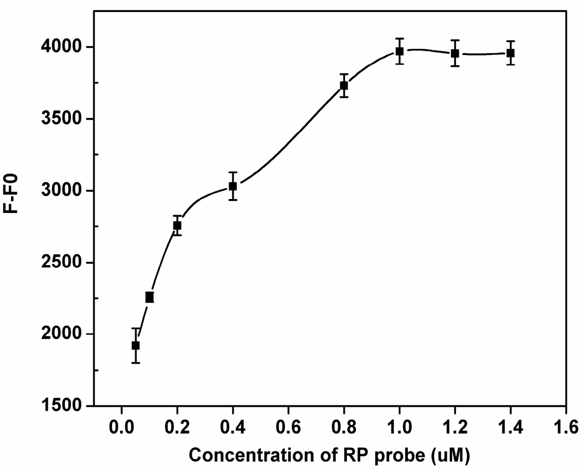

Figure S1. Effect of the concentration of the RP probe on the biosensor response, Conditions: C_{BPA} = 1.0 μM, $C_{Circle\ DNA}$ = 100 nM, C_{GHP} = 25 μM. $C_{Exo\ III}$ = 100 U, C_{ZnPPIX} = 20 μM. RCA reaction time 1.5 h. Error bars show the standard deviation of three experiments.

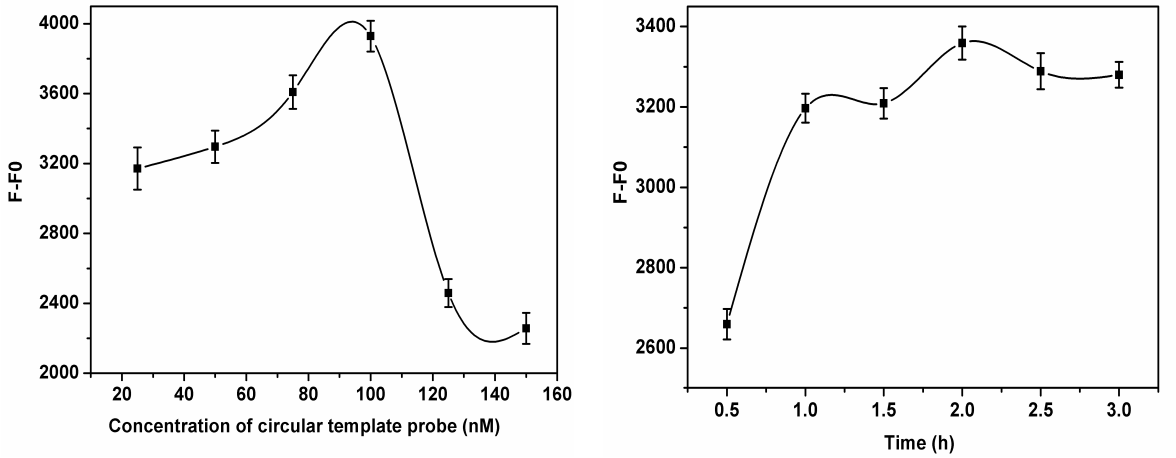

(**a**) (**b**)

Figure S2. (**a**) Effect of the concentration of the circle template probe on the biosensor response, Conditions: C_{BPA} = 1.0 μM, C_{RP} = 1.0 μM, C_{GHP} = 25 μM, $C_{Exo\ III}$ = 100 U, C_{ZnPPIX} = 20 μM, RCA reaction time 1.5 h. Error bars show the standard deviation of three experiments; (**b**) Effect of the RCA reaction time on the biosensor response, Conditions: C_{BPA} = 1.0 μM, C_{RP} = 1.0 μM, $C_{Circle\ DNA}$ = 100 nM, C_{GHP} = 25 μM, $C_{Exo\ III}$ = 100 U, C_{ZnPPIX} = 20 μM. Error bars show the standard deviation of three experiments.

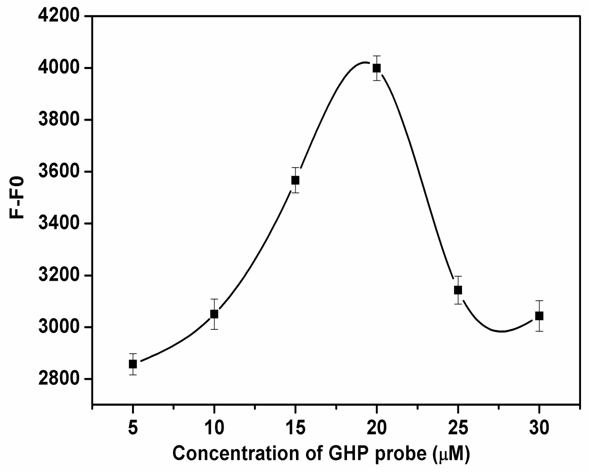

Figure S3. Effect of the concentration of the GHP probe on the biosensor response, Conditions: C_{BPA} = 1.0 μM, C_{RP} = 1.0 μM, $C_{Circle\ DNA}$ = 100 nM, $C_{Exo\ III}$ = 100 U, C_{ZnPPIX} = 20 μM. RCA reaction time 2 h. Error bars show the standard deviation of three experiments.

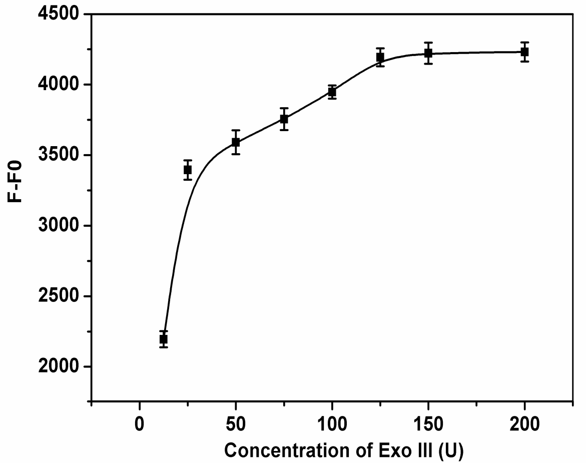

Figure S4. Effect of the concentration of Exo III on the biosensor response, Conditions: C_{BPA} = 1.0 μM, C_{RP} = 1.0 μM, $C_{Circle\ DNA}$ = 100 nM, C_{GHP} = 20 μM, C_{ZnPPIX} = 20 μM. RCA reaction time 2 h. Error bars show the standard deviation of three experiments.

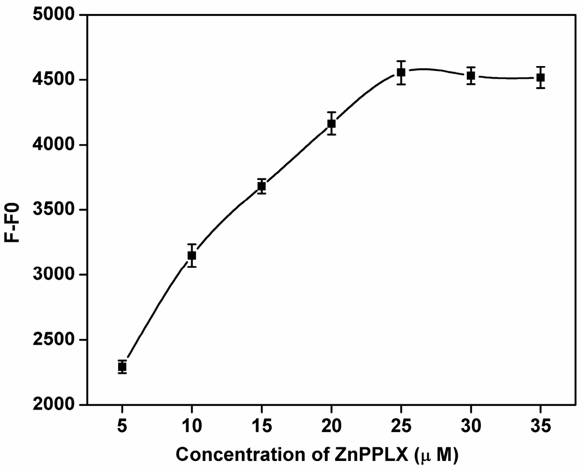

Figure S5. Effect of the concentration of ZnPPIX on the biosensor response, Conditions: C_{BPA} = 1.0 μM, C_{RP} = 1.0 μM, $C_{Circle\ DNA}$ = 100 nM, C_{GHP} = 20 μM, $C_{Exo\ III}$ = 125 U, RCA reaction time 2 h. Error bars show the standard deviation of three experiments.

Table S1. Comparison of the previous methods for BPA detection.

Signal Readout	Amplification	LOD	Linear Range (M)	Real Sample	Ref.
electrochemistry	−	2.0×10^{-8} M	$0.1–1.0 \times 10^{-6}$	PC bottles	[1]
chemiluminescence	−	3.1×10^{-7} M	$8 \times 10^{-7}–1 \times 10^{-5}$	PC bottles	[2]
colorimetric	−	0.1×10^{-9} M	−	water samples	[3]
fluorescence	−	1.4×10^{-9} M	−	water samples	[4]
fluorescence	−	1.86 ng/mL	10–80 ng/mL	water samples	[5]
fluorescence	−	0.008 ng/mL	0.02–5 ng/mL	water samples	[6]
fluorescence	+	5×10^{-15} M	$10^{-14}–10^{-9}$M	water samples	[7]
fluorescence	+	5.4×10^{-17} M	$10^{-16}–10^{-9}$ M	water samples PC bottles	This work

The "−" in the table represents with the amplification process, and the "+" in the table represents without the amplification process.

Table S2. The recovery results of BPA in the different sample matrixes.

Sample	[a]Spiked (pM)	[a]Found (pM)	RSD (%)	Recovery (%)
Tap water 1	0	0.005	4.2	-
Tap water 2	0.005	0.0094	5.1	94.0
Tap water 3	0.01	0.016	3.9	106.7
Tap water 4	0.015	0.022	5.9	110.0
Mineral water 1	0	−	−	−
Mineral water 2	0.005	0.0044	3.5	94.4
Mineral water 3	0.01	0.0098	4.8	96.2
Mineral water 4	0.015	0.0156	5.5	102.7
plastic bottle 1	0	0.036	3.9	−
plastic bottle 2	0.036	0.076	4.4	105.6
plastic bottle 3	0.072	0.112	5.6	103.7
plastic bottle 4	0.108	0.149	6.1	103.4

[a] The data reported in the table represents the average of three measurements; "−" Not detected.

References

1. Kou, L.; Liang, R.; Wang, X.; Chen, Y.; Qin, W. Potentiometric sensor for determination of neutral bisphenol A using a molecularly imprinted polymer as a receptor. *Anal. Bioanal. Chem.* **2013**, *405*, 4931–4936.
2. Wang, S.; Wei, X.; Du, L.; Zhuang, H. Determination of bisphenol A using a flow injection inhibitory chemiluminescence method. *Luminescence* **2005**, *20*, 46–50.
3. Mei, Z.; Chu, H.; Chen, W.; Xue, F.; Liu, J.; Xu, H.; Zhang, R.; Zheng, L. Ultrasensitive one-step rapid visual detection of bisphenol A in water samples by label-free aptasensor. *Biosens. Bioelectron.* **2013**, *39*, 26–30.
4. Rodriguez-Mozaz, S.; Alda, M.L.; Barcelo, D. Analysis of bisphenol A in natural waters by means of an optical immunosensors. *Water Res.* **2005**, *39*, 5071–5079.
5. Li, Y.; Xua, J.; Wang, L.; Huang, Y.; Guo, J.; Cao, X.; Shen, F.; Luo, Y.; Sun, C. Aptamer-based fluorescent detection of bisphenol A using nonconjugated gold nanoparticles and CdTe quantum dots. *Sens. Actuators B* **2016**, *222*, 815–822.
6. Hua, K.; Yin, H.; Liu, L.; Xu, L.; Ma, W.; Xu, C. Asymmetric plasmonic aptasensor for sensitive detection of bisphenol A. *ACS Appl. Mater. Interfaces* **2014**, *6*, 364–369.
7. Chen, J.; Zhou, S. Label-free DNA Y junction for bisphenol A monitoring using exonuclease III-based signal protection strategy. *Biosens. Bioelectron.* **2016**, *77*, 277–283.
